# Clinical Application of MAGiC Method in Distinguishing Between Pituitary Adenoma and Rathke’s Cleft Cyst

**DOI:** 10.3390/diagnostics15131607

**Published:** 2025-06-25

**Authors:** Ra Gyoung Yoon, Boeun Lee, Moon jung Hwang, Soo Jeong Park

**Affiliations:** 1 Department of Radiology, Nowon Eulji University Hospital, Eulji University College of Medicine, Seoul 01830, Republic of Korea; yoonrg@gmail.com; 2 Department of Radiology, Ewha Womans University College of Medicine, Seoul 07985, Republic of Korea; 3 Advanced Medical Imaging Institute, Korea University Anam Hospital, Seoul 02841, Republic of Korea; 4 Department of Neurosurgery, Ewha Womans University College of Medicine, Seoul 07985, Republic of Korea; parksoojeong22@ewha.ac.kr

**Keywords:** magnetic resonance image compilation, magnetic resonance imaging, pituitary adenoma, Rathke’s cleft cyst

## Abstract

**Background:** Differentiating pituitary adenoma (PA) from Rathke’s cleft cyst (RCC) on magnetic resonance imaging (MRI) remains challenging due to overlapping imaging features such as the cystic appearance, and location within the sellar region. A magnetic resonance image compilation (MAGiC) sequence can simultaneously acquire R1 and R2 relaxation rates. This study evaluated the diagnostic performance of MAGiC-driven imaging parameters for distinguishing PA from RCC. **Methods:** In total, 108 patients (54 each with PA or RCC) who underwent MRI using the MAGiC sequence were included in this study. The R1 and R2 relaxation rates were measured from the regions of interest identified in the MAGiC images. The relaxation rates between the PA and RCC groups were compared and diagnostic performance was assessed. **Results:** The relaxation rates of PA and RCC differed significantly. PA exhibited lower R1 (0.71 vs. 1.31, *p* < 0.001) and R2 (13.62 vs. 11.38, *p* = 0.029) relaxation rates than RCC. The R1 relaxation rate demonstrated superior diagnostic performance, with an area under the curve (AUC) of 0.74 (95% confidence interval [CI]: 0.65–0.82), surpassing the R2 rate (AUC 0.62; 95% CI: 0.52–0.71). The optimal threshold for R1 was determined to be 0.82, which provided a sensitivity of 79.6% and specificity of 74.1% (*p* < 0.001), whereas the optimal threshold for R2 was 14.89 (*p* = 0.030). **Conclusions:** MAGiC-driven parameters, particularly the R1 relaxation rate, show promise for improving the differentiation between PA and RCC. These findings suggest the potential for the broader application of MAGiC imaging in clinical practice to improve diagnostic accuracy.

## 1. Introduction

Pituitary adenomas (PAs) and Rathke’s cleft cysts (RCCs) are the most common conditions in the sellar and suprasellar region [1]. Distinguishing between these two conditions is crucial because of their different treatment strategies and potential clinical outcomes as PA is a neoplasm whereas RCC is a non-neoplastic cystic lesion derived from the remnants of the Rathke’s pouch [1,2,3]. RCCs often require minimal surgical intervention, such as in cyst content evacuation and the partial resection of the cyst wall, and can even be observed in small, asymptomatic RCC, whereas cystic PAs require complete surgical excision [1,2,4,5].

Despite the clinical significance of distinguishing between these two conditions, differentiating RCC from cystic PA using magnetic resonance imaging (MRI) can be challenging because of the variable imaging findings influenced by the cyst contents [6,7]. To date, conventional MRI sequences for the pituitary gland, including T1-weighted, T2-weighted, and dynamic contrast-enhanced (CE) images, have demonstrated variable diagnostic performances for differentiating between these two conditions [3,4,7,8,9]. Recent studies have aimed to improve the diagnostic accuracy through various imaging-based analyses. For example, a diagnostic model proposed by Park et al. identified key imaging features, such as fluid–fluid levels, hypointense rims on T2-weighted images, septations, locations, and intracystic nodules, for differentiating between cystic PA and RCC [8]. Although a recent study suggested that a typical ‘donut-like’ enhancement pattern along the inner margin of cysts on CE-3D T2-fluid-attenuated inversion-recovery (FLAIR) imaging could be a diagnostic marker for cystic PA, unresolved issues remain regarding the qualitative imaging analyses due to interobserver variability and inconsistency [9].

The magnetic resonance imaging compilation (MAGiC) sequence is an advanced synthetic MRI technique that enables the simultaneous quantification of multiple relaxation parameters [10]. The MAGiC sequence utilized in our study was based on methodological developments originally proposed by Warntjes et al. [11,12], who introduced a rapid and clinically feasible technique for the simultaneous quantification of T1, T2, and proton density (PD). Their frameworks, QRAPTEST and QRAPMASTER, address the key limitations of earlier methods, such as long scan times, intersequence misregistration, and parameter interdependence, by employing a saturation-recovery and multi-echo acquisition scheme with an intrinsic B1 inhomogeneity correction. These principles enable the generation of both quantitative maps and synthetic contrast-weighted images from a single acquisition. Building on these foundations, the MAGiC sequence was implemented and commercialized by GE Healthcare [10]. It allows for the simultaneous acquisition of comprehensive quantitative data, thereby providing reliable, multiparametric insights into the signal intensity characteristics of lesions on MRI [10,13]. A recent study demonstrated the clinical utility and diagnostic ability of synthetic MRI using MAGiC sequences for detecting multiple sclerosis (MS) plaques [14].

In this study, we investigated the potential diagnostic applications of the MAGiC sequence for distinguishing PA from RCC through quantitative analyses, including R1 and R2 relaxation times derived from the MAGiC sequence. This was the first study to evaluate the diagnostic performance of MAGiC-sequence-based quantitative MRI parameters in differentiating between PA and RCC.

## 2. Materials and Methods

This retrospective study was approved by the institutional review board of Ewha Womans University Seoul Hospital, Seoul, Republic of Korea; the requirement for informed consent was waived. The study design and report format are in accordance with the Strengthening the Reporting of Observational Studies in Epidemiology (STROBE) guidelines.

### 2.1. Study Population

We retrospectively assessed consecutive patients who were radiologically or pathologically diagnosed with PA or RCC between December 2022 and December 2023 at the Ewha Womans University Seoul Hospital. The inclusion criteria for this study were as follows: patients with (a) a MAGiC sequence at initial diagnosis, (b) a lesion size > 5 mm, and (c) age > 20 years. In total, 108 patients were included in this study, comprising 54 patients with PA and 54 with RCC.

### 2.2. Image Acquisition and Analysis

All MRI studies were performed using the SIGNA Architect 3-Tesla MRI system (GE Healthcare, Milwaukee, WI, USA) with a 48-channel head coil (AIR^TM^ Coil; GE Healthcare).

The MAGiC sequence uses four automatically calculated saturation delays with two echo times of 21.4 and 85.7 ms, a repetition time of 4500–4550 ms, a field-of-view (FOV) of 240 mm, a phase FOV of 0.8, a matrix of 320 × 256, a slice thickness/spacing of 4/1 mm (acq. voxel size 0.8 × 0.9 × 4), 30 slices to cover the whole brain, a bandwidth of 22.73 KHz, an echo train length of 12, a number of averages of 1, and ASSET, a parallel acceleration factor of 2. The scan time of the synthetic MAGiC sequence was approximately 5 min and 42 s. A neuroradiologist (B.L., with 10 years of experience) reviewed all images and was blinded to the initial diagnosis.

**Figure 1:**
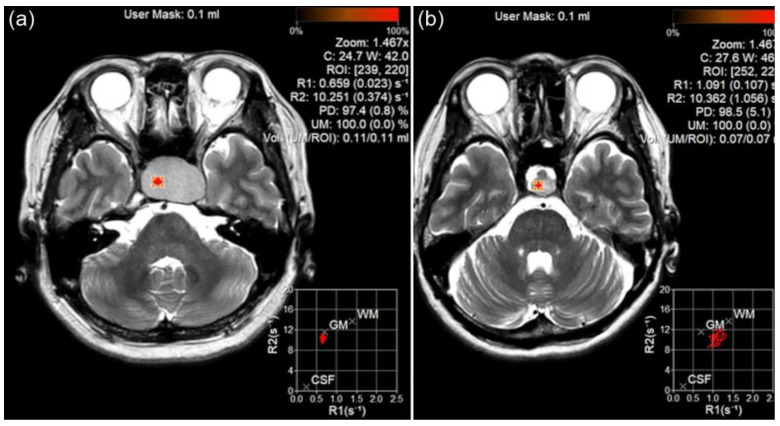
Region of interest (ROI) measurement for pituitary adenoma (PA) and Rathke’s cleft cyst (RCC) using magnetic resonance imaging compilation (MAGiC) image. The ROI was placed on PA (**a**) and RCC (**b**), and T1 and T2 relaxation rates were calculated.

Quantitative analyses (measurements of R1 and R2 relaxation rates) were conducted by a neuroradiologist (B.L.) using circular region-of-interest (ROI) measurements of MAGiC images. The neuroradiologist manually placed multiple round or oval ROIs in the lesions and the median value of the multiple ROIs was used (Figure 1). The radiologist placed a large ROI covering the entire lesion and designated multiple smaller ROIs for each individual component of heterogeneous lesions. This approach was adopted to enhance the representation of the overall quantitative values of the lesions.

### 2.3. Statistical Analyses

Each patient’s clinicopathological features of PA or RCC were analyzed using Fisher’s exact test for categorical variables and Mann–Whitney U test for continuous variables, including age and maximal nodule diameter, and are presented as median and interquartile range (IQR) values.

Owing to nonparametric distribution, the Mann–Whitney U test was used to compare the R1 and R2 relaxation rates between PA and RCC. A receiver operating characteristic (ROC) curve was used to evaluate the usefulness of R1 and R2 relaxation rates in distinguishing between PA and RCC. The area under the ROC curve (AUC) and the optimal threshold for the R1 and R2 relaxation rates were calculated according to Youden’s index [15]. Statistical analyses were performed using SPSS version 25 for Windows (IBM Corp., Armonk, NY, USA). Statistical significance was set at *p* < 0.05.

## 3. Results

### 3.1. Patients’ Clinical Characteristics

This study included 54 patients each with PA or RCC. The clinical characteristics of the patients are summarized in Table 1. The median size (maximum diameter) was larger in the PA group than in the RCC group (19.1 vs. 13.2 mm; *p* < 0.001). There were no significant differences in the median age (*p* = 0.169) or number of female patients (*p* = 0.330) between the two groups.

### 3.2. Comparison of R1/R2 Relaxation Rates

Table 2 presents the R1 and R2 relaxation rates in the PA and RCC groups. The PA group showed significantly lower R1 (0.71 vs. 1.31; *p* < 0.001) and R2 (13.62 vs. 11.38; *p* = 0.029) relaxation rates than the RCC group.

### 3.3. Comparison of Diagnostic Performance Using R1 and R2 Relaxation Rates

**Figure 2:**
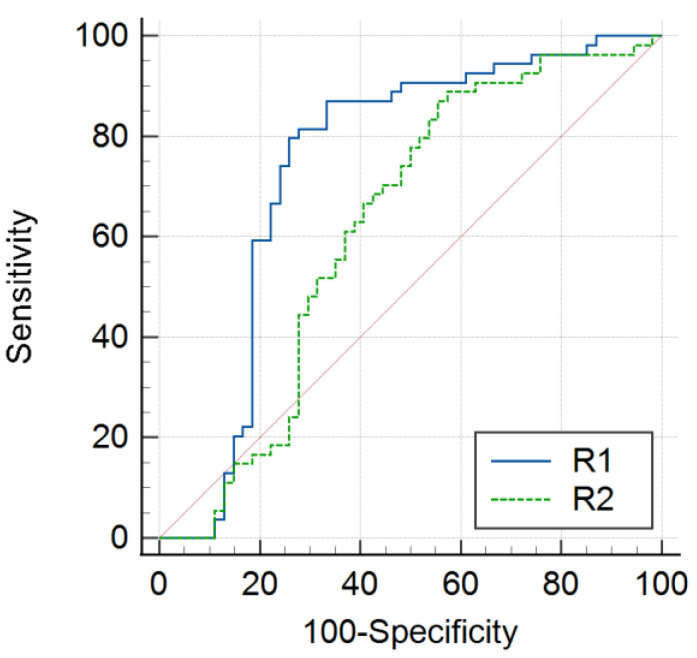
Comparison of R1 and R2 receiver operating characteristic (ROC) curves between patients with PA or RCC. The blue and green lines represent the R1 and R2 ROC curves, respectively, between the PA and RCC groups. When comparing the R1 and R2 curves, the sensitivity of the R1 relaxation rate was higher for the differential diagnosis of PA or RCC.

The R1 relaxation rate indicated better diagnostic performance for differentiating PA from RCC (AUC 0.74; 95% confidence interval [CI]: 0.65–0.82) than the R2 relaxation rate (AUC 0.62; 95% CI: 0.52–0.71). The optimal threshold for the R1 relaxation rate was 0.82, with a sensitivity of 79.6% and specificity of 74.1% (*p* < 0.001) (Table 3; Figure 2).

## 4. Discussion

We demonstrated that the PA group exhibited significantly lower R1 and R2 relaxation rates than the RCC group. Notably, the R1 relaxation rate showed superior diagnostic performance than the R2 relaxation rate, suggesting the superiority of the R1 relaxation rate in differentiating between PA and RCC. To the best of our knowledge, this was the first study to demonstrate the diagnostic application of R1 and R2 relaxation rates acquired via the MAGiC sequence for this purpose, providing quantitative analysis to differentiate PA from RCC.

The MAGiC sequence is a highlighted technique because of its ability to simultaneously acquire both R1 and R2 relaxation rates within a single scan by fitting the R1, R2, and PD values [10,11,12,13,16]. This innovative approach significantly simplifies traditionally complex and time-consuming procedures by generating a wide array of multicontrast magnetic resonance images from a single acquisition. The sequence’s multidynamic multi-echo method enables the efficient and reliable acquisition of comprehensive quantitative data, including T1, T2, and PD, making MAGiC a robust and versatile tool for clinical imaging, especially when precise and reproducible data are crucial [10,11,12,13,14]. Several previous studies have demonstrated the successful application of the MAGiC sequence under various conditions such as in detecting MS plaques, discriminating normal and abnormal tissues in the brain, planning radiotherapy for cancer, and assessing activity in sacroiliitis [14,17,18,19]. Therefore, we hypothesized that quantitative parameters derived from the MAGiC sequence could be used to differentiate between PA and RCC.

Our study was the first attempt to explore the diagnostic application of synthetic MRI-based quantitative parameters to differentiate between neoplastic and benign lesions. Hagiwara et al. attempted to use synthetic MRI methods to detect MS plaques and identified more MS plaques than conventional MRI with a comparable acquisition time [14]. They also found that synthetic double-inversion recovery images provided superior contrast for MS plaques than conventional images [14]. However, they used qualitative analysis methods, such as lesion-to-white matter (WM) contrast and the lesion-to-WM contrast-to-noise ratio, and not quantitative values such as the relaxation time from the MRI sequence. Jiang et al. demonstrated that MAGiC T1 and T2 values could be useful for differentiating patients with active sacroiliitis from inactive and control groups [17]. Another recent pilot study using quantitative MRI maps obtained with MAGiC showed clear differences in quantitative T1 and T2 values between post-treatment tissue abnormalities and healthy tissues in treated patients with glioma; quantitative T1 and T2 values also predicted abnormal tissue enhancement [18]. An advance in our study was that we directly obtained the R1 and R2 relaxation rates, and the R1 relaxation rate was more useful than the R2 relaxation rate in distinguishing between PA and RCC. Overall, our results and those of previous reports suggest that quantitative analyses using the MAGiC sequence can be expanded as a pretreatment imaging strategy in routine clinical practice to differentiate intracranial lesions.

In our study, PA demonstrated significantly lower R1 and R2 relaxation rates than RCC. Because R1 and R2 are reciprocals of the T1 and T2 relaxation times, respectively, these findings suggest that PA has longer T1 and T2 relaxation times than RCC. The observed differences may reflect distinct tissue compositions. RCC is a cystic lesion often filled with proteinaceous or mucoid fluid, mucopolysaccharides, and other macromolecules, which may enhance local magnetic field inhomogeneity, thereby promoting more efficient longitudinal relaxation and resulting in shorter T1 and higher R1 values [1,4,6,8]. This phenomenon is consistent with previous reports indicating that protein-rich cystic fluids exhibit an elevated R1 (shorter T1) compared to water or less proteinaceous environments [8,20,21]. Ijare et al. also suggested that the unique biochemical composition of RCC cyst fluid, including the presence of glycosaminoglycans (GAGs), increases fluid viscosity and alters relaxation properties using proton magnetic resonance spectroscopy [22]. These macromolecules can further promote T1 shortening by reducing free water mobility and increasing interactions between water molecules and cyst contents. In contrast, although PA is a solid tumor, our study demonstrated a lower R1 relaxation rate in PA, which could be attributed to the complex extracellular water content and microstructural heterogeneity, all of which may change water mobility and reduce relaxation efficiency, leading to a prolonged T1 and lower R1. For R2, RCC showed a slightly higher R2 relaxation rate compared to PA, which was consistent with the known biochemical properties of RCC. Protein-rich cystic fluids, including mucopolysaccharides and GAGs, are known to increase local magnetic field inhomogeneity and restrict water mobility, thereby accelerating transverse magnetization dephasing, resulting in shorter T2 relaxation times and, accordingly, higher R2 values. In contrast, the PA group in our study demonstrated a slightly lower R2 value. This finding was somewhat counterintuitive as PA is generally expected to show a higher R2 due to its solid structure. Our findings may reflect the influence of diverse tissue components such as the extracellular water content and localized magnetic field properties. Interestingly, both RCC and PA groups showed substantial variability in R2 relaxation rates compared to R1. This may have reflected heterogeneous biochemical and structural compositions within each lesion type that contribute to variation in R2. In particular, RCC may contain a wide range of cystic fluid characteristics, contributing to a broader distribution of the R2 relaxation rate observed. Accordingly, the interpretation of R2 should be approached with caution due to the small sample size, absence of histopathologic correlation or biochemical validation, and preliminary nature of our study. Future investigations incorporating detailed cystic fluid analysis and pathological correlation are needed to clarify the underlying tissue-based mechanisms.

RCC is a relatively common condition of the pituitary gland and is mostly indolent [1]. Although asymptomatic RCCs can often be managed conservatively owing to their natural course, which includes a decrease in size and spontaneous resolution, some patients with RCC require surgery when diagnostic challenges arise in distinguishing between cystic PA and RCC on MRI [23,24,25]. Moreover, the surgical techniques and outcomes for RCC differ from those for other pituitary neoplasms [4,5,26,27]. Therefore, distinguishing between PA and RCC preoperatively is crucial for planning treatment strategies and predicting outcomes [2,4]. In a recent study, Tavakol et al. introduced a classification scheme for cystic sellar lesions based on clinical and imaging features; symptomatic hyperprolactinemia, obesity, higher cystic lesion types, and fluid–fluid levels predicted cystic PA with high diagnostic accuracy [28]. Regarding the imaging characteristics, Azuma et al. suggested that donut-like enhancement along the inner margin of the cyst on CE-3D T2-FLAIR was useful to differentiate cystic PA from RCC [9]. However, RCC may show wall enhancement due to a partial volume-averaging effect of the surrounding normal pituitary gland, inflammation, or squamous metaplasia of the cyst wall [6,29]. Park et al. proposed a diagnostic model using MRI to distinguish cystic PA from RCC. Their model showed that the presence of a fluid–fluid level, septation, an off-midline location, and an intra-cystic nodule could enhance the diagnostic accuracy for differentiating cystic PA from RCC [8]. Another recent study explored the use of ex vivo 1H-NMR spectroscopy to exclusively identify GAGs in RCC and suggested that GAGs are potential diagnostic markers [22]. However, challenges remain regarding the variable signal intensity of RCC, which depends on the cyst content, including proteins, cholesterol, and mucopolysaccharides [6,7]. To overcome the limitations of previous qualitative analyses, we sought to differentiate PA and RCC using quantitative values derived from the MAGiC sequence. Our results showed the MAGiC sequence can provide reproducible and quantitative values in a single sequence, thereby facilitating the use of quantitative parameters to differentiate between PA and RCC, even by less experienced researchers.

Our study had some limitations. First, this was a retrospective study with data collected from a single institution, Ewha Womans University Seoul Hospital. This may have introduced a selection bias, which may limit the generalizability of our findings. Additionally, our study population was relatively small, with only 54 patients in each group (PA and RCC), which may have affected statistical power. Third, ROI placement was performed by a single neuroradiologist, which resulted in a lack of interobserver agreement. However, because the R1 and R2 relaxation rates are quantitative and absolute values, the effect of interobserver variability on the measurement was less significant. Finally, we focused on a preliminary investigation to determine the potential diagnostic applications of MAGiC-based quantitative values for intracranial mass lesions, including PA and RCC. Therefore, the results inherently limit our ability to draw firm conclusions regarding diagnostic performance as they were not intended to demonstrate diagnostic superiority or to be directly compared to previous studies. To develop diagnostic decision models, further prospective studies with larger populations and external validation are essential to validate our findings and enhance the clinical applicability of the MAGiC sequence.

## 5. Conclusions

This study highlighted the clinical utility of quantitative parameters derived from the MAGiC sequence for distinguishing between PA and RCC. Our results demonstrate that R1 relaxation rates provide superior diagnostic accuracy than R2 rates, advocating the broader application of MAGiC-based quantitative MRI in clinical practice, irrespective of the operator’s level of radiologic expertise. This sequence enables the rapid and simultaneous acquisition of multiparametric quantitative data that may have the potential for implementation in clinical settings.

## Figures and Tables

**Table 1:** Clinical characteristics of patients with pituitary adenoma or Rathke’s cleft cyst.

Characteristics	PA (*n* = 54)	RCC (*n* = 54)	*p* Value
Number of female patients	29 (53.7)	35 (64.8)	0.330
Age (years)	50.5 (42, 67)	48.0 (35, 61)	0.169
Maximal nodule diameter (mm)	19.1 (15.5, 25.3)	13.2 (9.2, 16.7)	<0.001

Data are expressed as medians with interquartile ranges in parentheses or as numbers with percentages in parentheses. PA: pituitary adenoma; RCC: Rathke’s cleft cyst.

**Table 2:** Comparison of R1/R2 parameters between patients with pituitary adenoma or Rathke’s cleft cyst.

Parameter	PA (*n* = 54)	RCC (*n* = 54)	*p* Value
R1 relaxation rate	0.71 (0.62, 0.81)	1.31 (0.81, 1.92)	<0.001
R2 relaxation rate	11.38 (10.0, 13.36)	13.62 (9.49, 19.19)	0.029

Data are expressed as medians with interquartile ranges in parentheses. PA: pituitary adenoma; RCC: Rathke’s cleft cyst.

**Table 3:** Comparison of diagnostic performance between R1 and R2 parameters in distinguishing between pituitary adenoma and Rathke’s cleft cyst.

Parameter	AUC	95% CI	Standard Error	Optimal Threshold	*p* Value
R1 relaxation rate	0.74	0.65, 0.82	0.05	0.82	<0.001
R2 relaxation rate	0.62	0.52, 0.71	0.06	14.89	0.030

AUC, area under the receiver operating characteristic curve; CI, confidence interval.

## Data Availability

The datasets used and/or analyzed during the current study are available from the corresponding author upon reasonable request.

## Abbreviations

The following abbreviations have been used in this manuscript:

PA	Pituitary adenoma
RCC	Rathke’s cleft cyst
MRI	Magnetic resonance imaging
MAGiC	Magnetic resonance imaging compilation
AUC	Area under the curve
CI	Confidence interval
CE	Contrast-enhanced
FLAIR	Fluid-attenuated inversion-recovery
PD	Proton density
MS	Multiple sclerosis
TEs	Two echo times
TR	Repetition time
FOV	Field of view
ROI	Region of interest
